# Predictability of orthodontic movement with orthodontic aligners: a retrospective study

**DOI:** 10.1186/s40510-017-0190-0

**Published:** 2017-11-13

**Authors:** Luca Lombardo, Angela Arreghini, Fabio Ramina, Luis T. Huanca Ghislanzoni, Giuseppe Siciliani

**Affiliations:** 1 Postgraduate School of Orthodontics, University of Ferrara, Via Fossato di Mortara, 44100 Ferrara, Italy; 2 Department of Biomedical Sciences and Health, University of Milan, Milan, Italy

**Keywords:** F22 aligner, Orthodontic movement, Movement accuracy, Predictability

## Abstract

**Background:**

The aim of this study was to evaluate the predictability of F22 aligners (Sweden & Martina, Due Carrare, Italy) in guiding teeth into the positions planned using digital orthodontic setup.

**Methods:**

Sixteen adult patients (6 males and 10 females, mean age 28 years 7 months) were selected, and a total of 345 teeth were analysed. Pre-treatment, ideal post-treatment—as planned on digital setup—and real post-treatment models were analysed using VAM software (Vectra, Canfield Scientific, Fairfield, NJ, USA). Prescribed and real rotation, mesiodistal tip and vestibulolingual tip were calculated for each tooth and, subsequently, analysed by tooth type (right and left upper and lower incisors, canines, premolars and molars) to identify the mean error and accuracy of each type of movement achieved with the aligner with respect to those planned using the setup.

**Results:**

The mean predictability of movements achieved using F22 aligners was 73.6%. Mesiodistal tipping showed the most predictability, at 82.5% with respect to the ideal; this was followed by vestibulolingual tipping (72.9%) and finally rotation (66.8%). In particular, mesiodistal tip on the upper molars and lower premolars were achieved with the most predictability (93.4 and 96.7%, respectively), while rotation on the lower canines was the least efficaciously achieved (54.2%).

**Conclusions:**

Without the use of auxiliaries, orthodontic aligners are unable to achieve programmed movement with 100% predictability. In particular, although tipping movements were efficaciously achieved, especially at the molars and premolars, rotation of the lower canines was an extremely unpredictable movement.

## Background

Since orthodontic aligners were launched on the market, they have been in growing demand among patients, especially adults, thanks to their aesthetic properties and clinical efficacy [1].

Although the idea of using consecutive clear thermoplastic appliances to align the teeth was first introduced by Kesling in 1946 [2], it was not until Align Technology (Santa Clara, CA, USA) launched the Invisalign system in 1998 that such appliances were prescribed on a large scale, thanks to their introduction of CAD/CAM technology into Orthodontics [3]. At first, aligners were marketed as an alternative to traditional fixed appliances in simple malocclusion cases such as slight crowding or minor space closure [4]. Over time, however, the range of malocclusion cases that can be treated by means of invisible aligners has widened. Clinical research has developed aligner-based solutions for even complex cases involving major rotation of the premolars, upper incisor torque, distalisation and/or extractive space closure [5].

That being said, there is as yet no consensus as to the predictability of aligner treatment in such large movements; although the aesthetic impact of aligners has been emphasised [6], few studies have yet been set up to investigate the effective capacity of aligners to achieve complex movements [7]. Indeed, the majority of articles published on aligner orthodontics have been case reports or series, reports on the use of a particular system, and expert opinions [3, 8, 9]. Furthermore, studies have concentrated on the market leader, Invisalign, even though many other competing systems have been developed since Align Technology’s patent expired. These alternative aligner systems differ from Invisalign in terms of construction material [10], production process, margin finishing and STL model precision, but perhaps the most influential difference is the professionals charged with executing treatment planning and setup (IT specialists, dental technicians or professional orthodontists) [11].

As regards treatment outcomes, Align Technology reports that roughly 20–30% of Invisalign patients require mid-course correction or post-alignment finishing in order to achieve the results prescribed on the setup [12]. This figure, however, contrasts with that reported by orthodontists, who indicate that the number of patients who require some unplanned correction or even recourse to fixed orthodontics, is closer to 70–80% [1, 13].

In fact, Kravitz [14] reported that Invisalign aligners had a mean accuracy of 41% in terms of achieving planned outcomes, with the most predictable movement being lingual contraction (47.1%), and the least predictable, extrusion (29.6%). In a systematic review of the literature, Rossini and Castroflorio confirmed that the most problematic movement for Invisalign was extrusion, followed by rotation [15].

However, these authors also emphasised the paucity of reliable literature on the subject, and the aim of this study was therefore to compare planned and achieved tipping and rotation in patients using F22 aligners (Sweden & Martina, Due Carrare, Italy) in order to provide data on their effective clinical predictability.

## Methods

### Sample selection

Sixteen adult Caucasian patients (6 males and 10 females, of mean age 28 years and 7 months) treated by means of F22 aligners at the University of Ferrara Postgraduate School of Orthodontics Clinic were retrospectively selected. Inclusion and exclusion criteria are reported in Table 1. Treatment staging, i.e. the maximum movement planned for each aligner, had been 2° rotation, 2.5° vestibulolingual and mesiodistal tip, and 0.2-mm linear displacement. No auxiliaries of any kind had been used (intermaxillary elastics, buttons, chains), although the use of F22 system Grip Points (attachments) and anterior and/or posterior stripping was allowed. Patients were instructed to wear their aligners for 22 h per day, excepting mealtimes and oral hygiene procedures. Aligners were replaced every 14 days.

**Table 1:** Inclusion and exclusion criteria

Inclusion criteria	Exclusion criteria
• Adult subjects > 18 years with permanent dentition • Complete dentition, or with 4 missing teeth at the most (third molars excluded) • No supernumerary teeth • No tooth shape anomalies • No dental rotation > 35° • No diastems > 5 mm • Crowding < 5 mm per arch	• Systemic pathologies • Ongoing pharmacological treatment able to influence orthodontic movement (e.g. prostaglandin inhibitors, biphosphonates) • Active periodontal disease • Treatments requiring extraction space closure

Pre-treatment, ideal post-treatment (according to setup) and real post-treatment digital models of the upper and lower jaws of each patient were analysed. Pre-treatment and post-treatment models were acquired using a Trios intraoral scanner (3Shape, Copenhagen, Denmark), and setups were constructed using Orthoanalyzer software (3Shape, Copenhagen, Denmark).

### Measurement of digital models

**Fig. 1:**
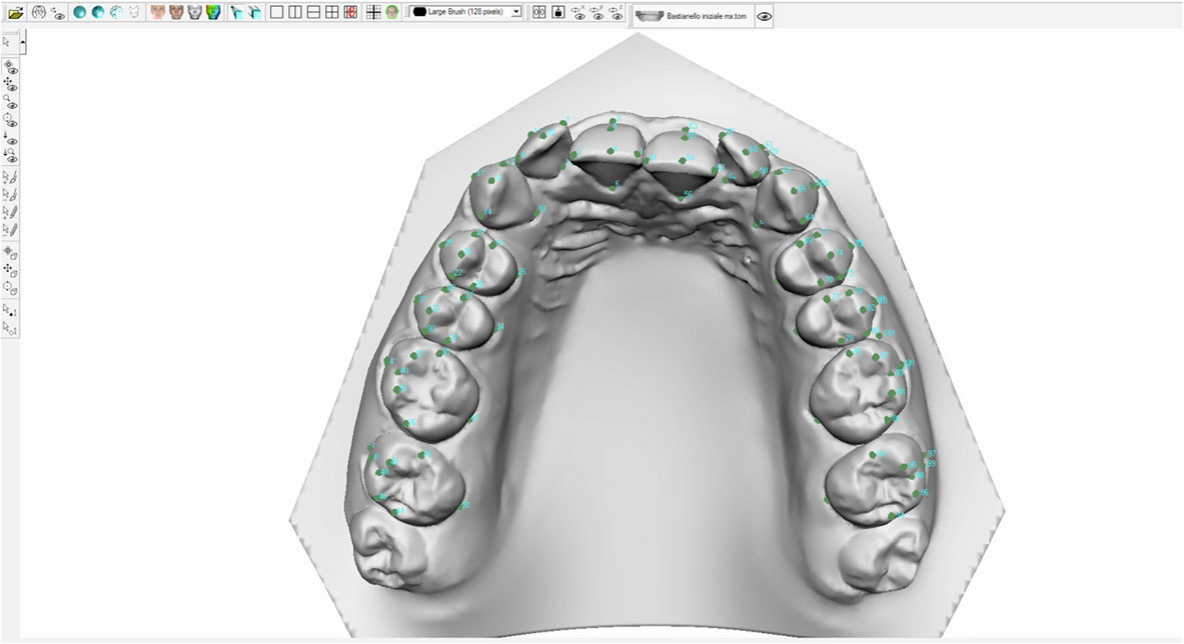
Positioning of the 100 reference points per arch (Upper jaw)

Digital models pertaining to each patient were analysed in .stl format by a single operator using VAM software (Vectra, Canfield Scientific, Fairfield, NJ, USA). This enabled the identification of anatomical reference points, planes and axes on the digital models, required, in turn, for calculation of the angulation, inclination and vestibular prominence of each tooth, as well as linear and angular measurements, for example, the intra-arch diameters [16]. Measurement was based on a method originally involving the identification of a total of 60 reference points per model (excluding second molars). However, in this case, we also included the second molars in the digital measurements, thereby expanding the number of reference points to 100 per model (Fig. 1).

**Fig. 2:**
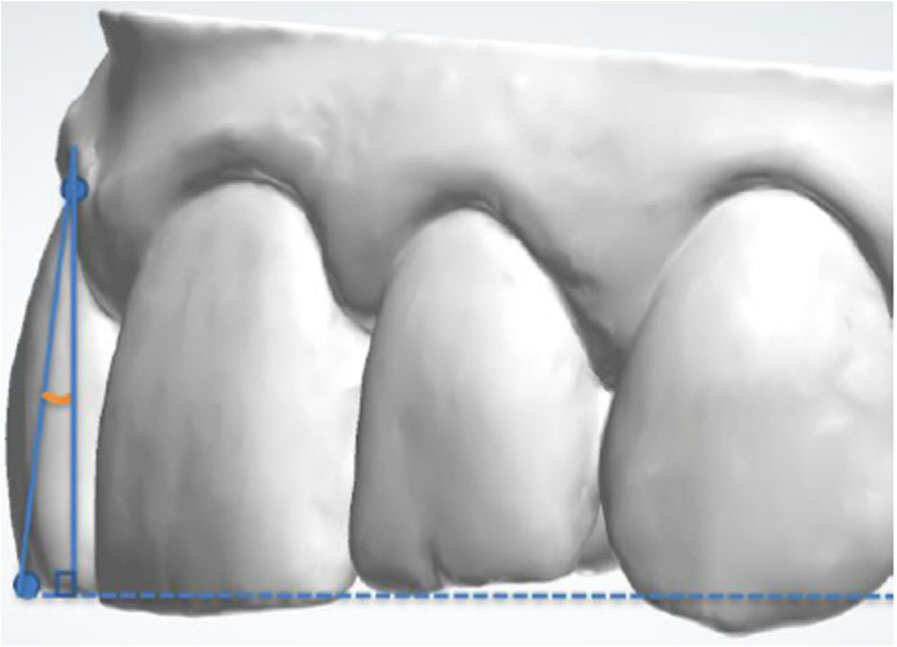
Vestibulolingual tipping: labiolingual inclination of the FACC with respect to the occlusal plane of reference

**Fig. 3:**
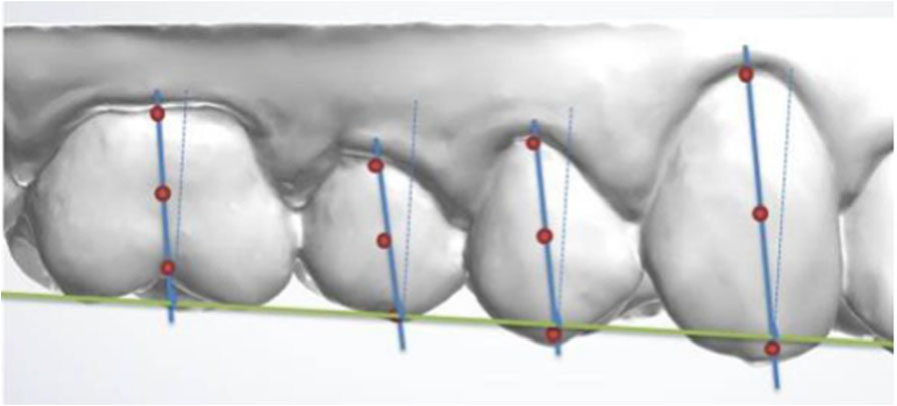
Mesiodistal tipping: mesiodistal inclination of the FACC with respect to the occlusal plane of reference

**Fig. 4:**
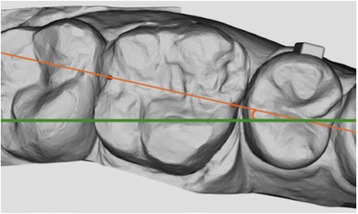
Rotation: the angle between the mesiodistal axis of the tooth and plane *y*

**Fig. 5:**
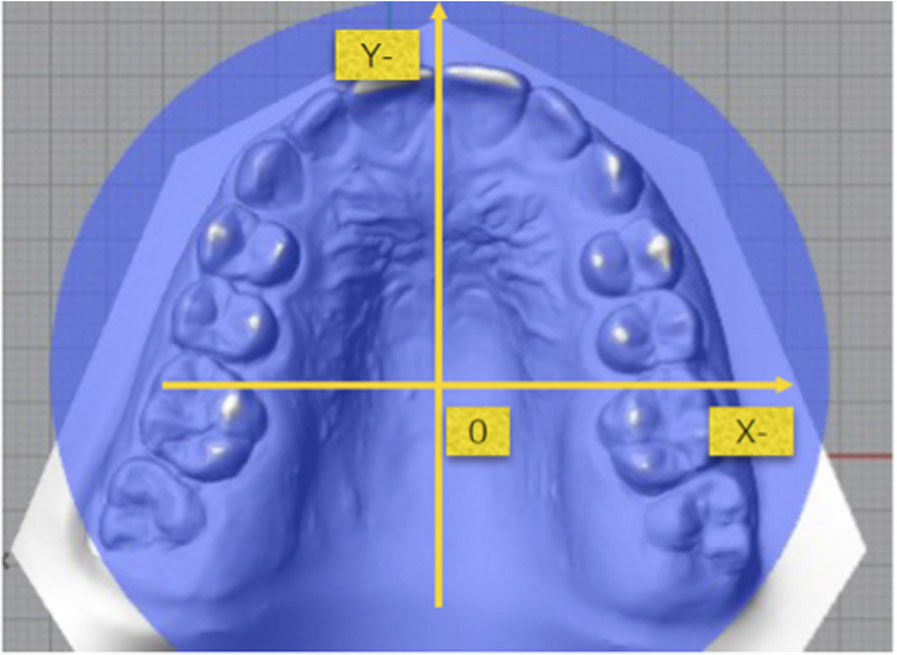
Occlusal plane of reference

Once the 100 reference points had been marked, their three-dimensional coordinates were extrapolated and exported, first into a .txt file, and then onto a dedicated spreadsheet provided with the software. This spreadsheet enabled extrapolation of the mesiodistal and vestibulolingual tip and rotation (Figs. 2, 3, and 4) of each tooth with respect to a 3D Cartesian grid based on the occlusal reference plane, which was obtained by means of the following points: (Fig. 5):

- Reference points at the mediovestibular cusps of teeth 16 in the maxilla and 46 in the mandible
- Reference points at the mediovestibular cusps of teeth 26 in the maxilla and 36 in the mandible
- The centroid of all occlusal points of the FACC (the facial axis of the clinical crown) of teeth 15, 14, 12, 11, 21, 22, 24 and 25 in the maxilla and 35, 34, 32, 31, 41, 42, 44 and 45 in the mandible; canines were excluded from this calculation as their occlusal FACC point is generally outside the occlusal plane identified by the other teeth.

One month after the 96 arches had been analysed, the analysis was repeated on 16 randomly selected digital models (8 upper and 8 lower arches). Dahlberg’s D was calculated in order to quantify the measurement error, and Student’s *t* test for paired data to identify any systematic error.

### Analysis of mean imprecision

The following calculations were made for each type of movement of each tooth in each patient:

- The absolute value of the prescription, i.e. the difference between ideal post-treatment and pre-treatment measurements, to identify the total programmed movement:

$$ \mid \mathrm{prescription}\mid =\mid \mathrm{ideal}\mathrm{posttreatment}-\mathrm{pretreatment}\mid $$

- The absolute value of the imprecision, i.e. the difference between ideal and real post-treatment measurements, to identify the difference between the actual post-treatment position of each tooth and the programmed movement:

$$ \mid \mathrm{imprecision}\mid =\mid \mathrm{ideal}\mathrm{posttreatment}-\mathrm{real}\mathrm{posttreatment}\mid $$

Absolute values were used for the prescription and imprecision parameters, as the direction of movement (clockwise vs. anticlockwise rotation, and lingual vs. vestibular or mesial vs. distal for the tip) was not taken into consideration. Prescription and imprecision values were grouped into eight categories (upper and lower incisors, canines, premolars and molars) and according to the three types of movement (mesiodistal tip, vestibulolingual tip and rotation).

The different types of tooth (incisors, canines, premolars and molars) were analysed separately because of the different anatomy of the crown and the root (both in shape and length), which inevitably results in a different response to the application of orthodontic forces, in particular, in the treatment with aligners. In addition, the upper jaw teeth were divided from the mandibular ones, due to the different type and compactness of the bone, which can greatly influence the orthodontic movement.

Movements with a prescription of less than 2° were excluded from the analysis. This sensitivity threshold was determined from the mean intra-operator error pertaining to measurements made using the VAM software, which has been previously published in the study validating the method [16].

Thus a database containing measurements of 345 teeth, subdivided into the following types, was obtained:

- 57 upper incisors
- 29 upper canines
- 53 upper premolars
- 37 upper molars
- 64 lower incisors
- 30 lower canines
- 52 lower premolars
- 23 lower molars

The Kolmogorov-Smirnov statistical test was used to determine the non-normal distribution of the mean imprecision, using the median as a measure of central tendency and the interquartile interval as an expression of its distribution. The Kruskal-Wallis H test (*p* < 0.05) was applied in cases of an imprecision of tooth/movement combination whose mean was different to the others.

### Analysis of movement accuracy

The following formula was used to quantify the accuracy of each movement for each tooth type with respect to the prescription:

$$ \mathrm{movement}\mathrm{accuracy}=\frac{\mathrm{realposttreatment}-\mathrm{initial}\mathrm{pretreatment}}{\mathrm{idealposttreatment}-\mathrm{initial}\mathrm{pretreatment}} $$

Thus, an index of the accuracy of each movement was obtained: the closer the value to 1, the more precise the dental movement achieved by the aligner series (100% of the prescription). The mean accuracy index, standard deviation and mean standard error were calculated for each type of movement in each tooth category, and Student’s *t* test for single samples (*p* < 0.05) was applied in cases in which the predictability of any type of movement/tooth was significantly different to 1, i.e. significantly lower than 100% of the prescription. Finally, F ANOVA (*p* < 0.05) and Bonferroni’s post hoc tests were applied if there was a statistically significant difference in the predictability among the different types of tooth movement.

## Results

Measurement method analysis confirmed that there were no systematic measurement errors in any of the mesiodistal tip, vestibulolingual tip or rotation values (Table 2). Table 3 shows the absolute values for the mean prescription and mean imprecision of each movement of each tooth, alongside the median, relative interquartile and statistical significance. In the upper arch, the least precise movement in terms of absolute values was incisor rotation (imprecision, 5.0° ± 5.3°), while the most precise movement was vestibulolingual tipping of the canines (imprecision, 2.5° ± 1.5°). In the lower arch, on the other hand, the least precision was recorded for premolar rotation (imprecision, 5.4° ± 5.8°), while the most precise movement was vestibulolingual tipping of the molars (imprecision, 1.3° ± 0.9°). In the upper arch, there was no statistically significant difference in imprecision between the different types of tooth movements, whereas in the lower arch the canines showed a significantly greater error in terms of rotation of the canines (6.9° ± 5.4°) with respect to the incisors (3.4° ± 2.5°) and molars (2.0° ± 1.8°). Likewise, the lower molar rotation imprecision was significantly more precise than the lower incisor rotation.

**Table 2:** Method analysis

Arch	Parameter	Vestibulolingual tip		Mesiodistal tip		Rotation	
		*D* Dahlberg	Systematic error *p* level	*D* Dahlberg	Systematic error *p* level	*D* Dahlberg	Systematic error *p* level
Upper arch	11	0.300	NS	0.390	NS	0.525	NS
	12	0.298	NS	0.979	NS	0.500	NS
	13	0.782	NS	0.656	NS	0.957	NS
	14	0.437	NS	0.783	NS	1.132	NS
	15	0.674	NS	0.814	NS	1.162	NS
	16	0.497	NS	0.081	NS	1.290	NS
	17	0.686	NS	1.014	NS	0.964	NS
	21	0.075	NS	0.274	NS	1.174	NS
	22	0.785	NS	0.292	NS	0.788	NS
	23	0.753	NS	0.433	NS	1.081	NS
	24	0.539	NS	1.159	NS	0.883	NS
	25	0.636	NS	0.715	NS	2.135	NS
	26	0.579	NS	0.097	NS	1.214	NS
	27	0.358	NS	1.254	NS	1.616	NS
	Mean	0.528		0.639		1.102	
Lower arch	31	0.658	NS	0.348	NS	0.551	NS
	32	0.474	NS	0.536	NS	0.773	NS
	33	0.445	NS	0.593	NS	0.926	NS
	34	0.882	NS	0.581	NS	0.965	NS
	35	0.334	NS	0.100	NS	0.800	NS
	36	1.119	NS	1.510	NS	1.314	NS
	37	0.954	NS	1.110	NS	1.527	NS
	41	0.338	NS	0.351	NS	0.540	NS
	42	0.810	NS	0.673	NS	1.275	NS
	43	0.423	NS	0.752	NS	1.233	NS
	44	0.877	NS	0.856	NS	1.305	NS
	45	0.824	NS	0.653	NS	1.432	NS
	46	1.131	NS	0.932	NS	1.389	NS
	47	0.960	NS	1.262	NS	1.468	NS
	Mean	0.731		0.733		1.107	

*NS* not significant

**Table 3:** Mean prescription and mean imprecision values

		*N*	Mean prescription(°)	SD	Mean imprecision(°)	SD	Median	IQR	Significance.
Upper arch									
VL tip	Incisors	57	9.2	6.7	4.5	4.0	3.4	− 0.6	NS
	Canines	29	5.1	3.2	2.5	1.5	2.3	0.8	NS
	Premolars	53	5.1	3.4	3.1	2.6	2.1	− 0.5	NS
	Molars	37	3.9	1.4	2.9	2.2	2.5	0.3	NS
MD tip	Incisors	57	6.4	4.5	3.2	2.6	2.5	− 0.1	NS
	Canines	29	4.7	2.8	2.8	2.2	2.6	0.4	NS
	Premolars	53	4.6	3.3	3.6	2.3	3.9	1.6	NS
	Molars	37	4.5	1.6	3.4	2.3	3.4	1.1	NS
Rot.	Incisors	57	10.8	9.3	5.0	5.3	3.7	− 1.6	NS
	Canines	29	6.5	4.6	4.3	2.8	3.6	0.8	NS
	Premolars	53	7.0	6.7	3.5	3.1	2.9	− 0.2	NS
	Molars	37	7.2	4.8	4.8	4.6	4.4	− 0.2	NS
Lower arch									
VL tip	Incisors	64	5.9	2.1	2.9	2.6	2.3	− 0.3	NS
	Canines	30	7.2	5.0	3.5	2.8	3.1	0.3	NS
	Premolars	52	6.2	4.1	3.2	2.2	2.9	0.7	NS
	Molars	23	3.9	1.7	1.3	.9	1.9	1.0	NS
MD tip	Incisors	64	4.2	1.5	2.7	1.9	2.2	0.3	NS
	Canines	30	4.8	2.0	3.3	2.2	2.9	0.6	NS
	Premolars	52	5.4	4.7	3.4	2.6	3.1	0.5	NS
	Molars	23	6.3	3.7	4.3	3.0	3.5	0.5	NS
Rot.	Incisors	64	7.2	4.4	3.4	2.5	2.8	0.3	*
	Canines	30	12.4	10.0	6.9	5.4	5.5	0.1	*
	Premolars	52	7.3	6.0	5.4	5.8	3.7	− 2.1	NS
	Molars	23	4.6	2.8	2.0	1.8	1.4	− 0.4	*

*VL tip* vestibulolingual tip, *MD tip* mesiodistal tip, *Rot.* rotation, *SD* standard deviation, *IQR* interquartile range, *NS* not significant

*p < 0.05

Table 4 shows the mean accuracy, its standard deviation and standard error, and the statistical significance calculated for each type of tooth and tooth movement. In the upper arch, the inferential statistical analysis performed showed that neither the mesiodistal tip on the canines, premolars and molars, nor the rotation of the molars were significantly different from 1 (*p* < 0.05), chosen as the reference value to indicate 100% achievement of the planned movement. That being said, all other tooth movements displayed a predictability that was significantly lower than 100%. In contrast, in the lower arch, mesiodistal tipping and rotation of the canines and rotation of the incisors were significantly less accurate than 100%, while all other tooth movements achieved were not statistically different from the target movement.

**Table 4:** Accuracy of movements achieved

	N	Mean accuracy	Standard deviation	Mean standard error	Significance
Upper arch					
VL tip incisors	28	0.65	0.34	0.064714	*
VL tip canines	16	0.54	0.57	0.143044	*
VL tip premolars	32	0.70	0.81	0.142849	*
VL tip molars	16	0.52	0.53	0.133131	*
MD tip incisors	36	0.77	0.58	0.096078	*
MD tip canines	16	0.78	0.50	0.125380	NS
MD tip premolars	27	0.71	0.78	0.150417	NS
MD tip molars	22	0.98	0.98	0.217782	NS
Rot. incisors	45	0.61	0.29	0.042538	*
Rot. canines	25	0.62	0.66	0.131114	*
Rot. premolars	29	0.54	0.54	0.100854	*
Rot. molars	18	0.78	0.61	0.144458	NS
Lower arch					
VL tip incisors	35	0.86	0.65	0.109173	NS
VL tip canines	15	0.66	0.55	0.142351	*
VL tip premolars	29	0.90	0.82	0.151409	NS
VL tip molars	7	0.86	0.51	0.191882	NS
MD tip incisors	31	0.88	0.86	0.154196	NS
MD tip canines	18	0.87	0.82	0.193936	NS
MD tip premolars	33	0.97	0.97	0.168750	NS
MD tip molars	17	0.62	0.82	0.199778	NS
Rot. incisors	51	0.67	0.57	0.080357	*
Rot. canines	25	0.54	0.74	0.147841	*
Rot. premolars	36	0.83	1.38	0.229989	NS
Rot. molars	14	0.85	0.67	0.180257	NS

*VL tip* vestibulolingual tip, *MD tip* mesiodistal tip, *Rot.* rotation, *NS* not significant

*p < 0.05

Table 5 compares the mean accuracy among all tooth/movement combinations. This comparison revealed only one statistically significant difference. In other words, there was no greater precision statistically demonstrable in terms of one tooth movement with respect to another, with the exception of the lower incisors, whose rotation accuracy (0.40) was significantly lower than that of the lower premolars (0.87).

**Table 5:** Accuracy among tooth/movement combinations

Group/arch	Group/arch	Vestibulolingual tip			Mesiodistal tip			Rotation		
		Difference between means	Standard error	Significance	Difference between means	Standard error	Significance	Difference between means	Standard error	Significance
Incisors—upper arch	Incisors—lower arch	− .06361	.11235	NS	− .23697	.13106	NS	− .13364	.11270	NS
	Canines—upper arch	− .18249	.13887	NS	.02068	.16072	NS	− .24391	.13745	NS
	Canines—lower arch	− .07897	.14178	NS	− .22471	.15442	NS	− .27064	.13745	NS
	Premolars—upper arch	− .19907	.11467	NS	− .18541	.13618	NS	− .18593	.13121	NS
	Premolars—lower arch	− .18289	.11740	NS	− .28056	.12891	NS	− .4711025^*^	.12321	.005
	Molars—upper arch	− .10530	.13887	NS	− .36883	.14475	NS	− .13254	.15367	NS
	Molars—lower arch	.05389	.18725	NS	− .22751	.15741	NS	− .11360	.16863	NS
Incisors—lower arch	Incisors—upper arch	.06361	.11235	NS	.23697	.13106	NS	.13364	.11270	NS
	Canines—upper arch	− .11888	.13372	NS	.25765	.16466	NS	− .11027	.13453	NS
	Canines—lower arch	− .01537	.13675	NS	.01227	.15851	NS	− .13700	.13453	NS
	Premolars—upper arch	− .13546	.10838	NS	.05156	.14081	NS	− .05229	.12815	NS
	Premolars—lower arch	− .11928	.11127	NS	− .04359	.13379	NS	− .33746	.11995	NS
	Molars—upper arch	− .04170	.13372	NS	− .13186	.14912	NS	.00110	.15107	NS
	Molars—lower arch	.11749	.18347	NS	.00946	.16143	NS	.02004	.16626	NS
Canines—upper arch	Incisors—upper arch	.18249	.13887	NS	− .02068	.16072	NS	.24391	.13745	NS
	Incisors—lower arch	.11888	.13372	NS	− .25765	.16466	NS	.11027	.13453	NS
	Canines—lower arch	.10351	.15926	NS	− .24539	.18379	NS	− .02673	.15585	NS
	Premolars—upper arch	− .01658	.13568	NS	− .20609	.16876	NS	.05798	.15038	NS
	Premolars—lower arch	− .00040	.13800	NS	− .30124	.16295	NS	− .22719	.14345	NS
	Molars—upper arch	.07718	.15667	NS	− .38951	.17575	NS	.11137	.17033	NS
	Molars—lower arch	.23637	.20080	NS	− .24819	.18632	NS	.13031	.18394	NS
Canines—lower arch	Incisors—upper arch	.07897	.14178	NS	.22471	.15442	NS	.27064	.13745	NS
	Incisors—lower arch	.01537	.13675	NS	− .01227	.15851	NS	.13700	.13453	NS
	Canines—upper arch	− .10351	.15926	NS	.24539	.18379	NS	.02673	.15585	NS
	Premolars—upper arch	− .12010	.13866	NS	.03929	.16277	NS	.08471	.15038	NS
	Premolars—lower arch	− .10391	.14093	NS	− .05585	.15674	NS	− .20046	.14345	NS
	Molars—upper arch	− .02633	.15926	NS	− .14412	.17001	NS	.13810	.17033	NS
	Molars—lower arch	.13286	.20283	NS	− .00280	.18091	NS	.15704	.18394	NS
Premolars—upper arch	Incisors—upper arch	.19907	.11467	NS	.18541	.13618	NS	.18593	.13121	NS
	Incisors—lower arch	.13546	.10838	NS	− .05156	.14081	NS	.05229	.12815	NS
	Canines—upper arch	.01658	.13568	NS	.20609	.16876	NS	− .05798	.15038	NS
	Canines—lower arch	.12010	.13866	NS	− .03929	.16277	NS	− .08471	.15038	NS
	Premolars—lower arch	.01618	.11361	NS	− .09515	.13881	NS	− .28517	.13749	NS
	Molars—upper arch	.09377	.13568	NS	− .18342	.15363	NS	.05338	.16534	NS
	Molars—lower arch	.25296	.18490	NS	− .04210	.16562	NS	.07233	.17932	NS
Premolars—lower arch	Incisors—upper arch	.18289	.11740	NS	.28056	.12891	NS	.4711025^*^	.12321	.005
	Incisors—lower arch	.11928	.11127	NS	.04359	.13379	NS	.33746	.11995	NS
	Canines—upper arch	.00040	.13800	NS	.30124	.16295	NS	.22719	.14345	NS
	Canines—lower arch	.10391	.14093	NS	.05585	.15674	NS	.20046	.14345	NS
	Premolars—upper arch	− .01618	.11361	NS	.09515	.13881	NS	.28517	.13749	NS
	Molars—upper arch	.07758	.13800	NS	− .08827	.14723	NS	.33856	.15907	NS
	Molars—lower arch	.23677	.18660	NS	.05305	.15969	NS	.35750	.17356	NS
Molars—upper arch	Incisors—upper arch	.10530	.13887	NS	.36883	.14475	NS	.13254	.15367	NS
	Incisors—lower arch	.04170	.13372	NS	.13186	.14912	NS	− .00110	.15107	NS
	Canines—upper arch	− .07718	.15667	NS	.38951	.17575	NS	− .11137	.17033	NS
	Canines—lower arch	.02633	.15926	NS	.14412	.17001	NS	− .13810	.17033	NS
	Premolars—upper arch	− .09377	.13568	NS	.18342	.15363	NS	− .05338	.16534	NS
	Premolars—lower arch	− .07758	.13800	NS	.08827	.14723	NS	− .33856	.15907	NS
	Molars—lower arch	.15919	.20080	NS	.14132	.17273	NS	.01894	.19636	NS
Molars—lower arch	Incisors—upper arch	− .05389	.18725	NS	.22751	.15741	NS	.11360	.16863	NS
	Incisors—lower arch	− .11749	.18347	NS	− .00946	.16143	NS	− .02004	.16626	NS
	Canines—upper arch	− .23637	.20080	NS	.24819	.18632	NS	− .13031	.18394	NS
	Canines—lower arch	− .13286	.20283	NS	.00280	.18091	NS	− .15704	.18394	NS
	Premolars—upper arch	− .25296	.18490	NS	.04210	.16562	NS	− .07233	.17932	NS
	Premolars—lower arch	− .23677	.18660	NS	− .05305	.15969	NS	− .35750	.17356	NS
	Molars—upper arch	− .15919	.20080	NS	− .14132	.17273	NS	− .01894	.19636	NS

*NS* not significant

*p < 0.05

## Discussion

It is a common experience among clinicians that some tooth movements can be achieved more easily than others with aligners. However, the precise degree to which the achieved movements differ from the ideal movements planned using digital setups is difficult to quantify experimentally. First and foremost, it is necessary to identify stable structures within the oral cavity that can be used as reference points for superimposition of digital images. Among these, the palatine folds are the most frequently chosen [17], even though several studies have shown that their position and/or dimensions may vary in certain clinical conditions [18]. Furthermore, palatal structures may only be used as reference points in the upper jaw. This is one of the reasons why superimposition on stable teeth has been selected as the method of choice for evaluating the accuracy of Invisalign by several authors [14, 19, 20]. However, that method may only be used in cases in which orthodontic treatment involves the displacement of only some teeth; moreover, even if this is the case, collateral effects on the position of other teeth cannot be ruled out. Indeed, intrusion may occur due to the masticatory forces exerted when wearing aligners, and any teeth used as anchorage may be subject to reactionary displacement [20].

The method of tooth position measurement proposed by Huanca [16], on the other hand, is based on the occlusal plane as a point of reference. Calculated as the plane passing through the mesiovestibular cusps of the first molars and the centroid of the FACC of all of the other teeth, with the exception of canines, the occlusal plane is a reference that enables the measurement error due to tooth movement during orthodontic treatment to be minimised. Moreover, it is applicable to both arches in all individuals, and allows evaluation of orthodontic movement of all teeth, both anterior and posterior. What is more, the reliability of this method has been demonstrated for tooth movements greater than 2°, at which it displays no measurement or systematic error.

Using this method, we demonstrate that the mean accuracy of orthodontic movement provided by the F22 aligner is 73.6%, considering all movements in both anterior and posterior teeth, while it falls to 70.6% if only the anterior teeth are considered. Although derived from a different methodology, these figures appear to compare favourably with the 56 and 41% predictability achieved by Invisalign for anterior teeth reported by Nguyen and Cheng [21], and Kravitz et al. [14], respectively.

**Fig. 6:**
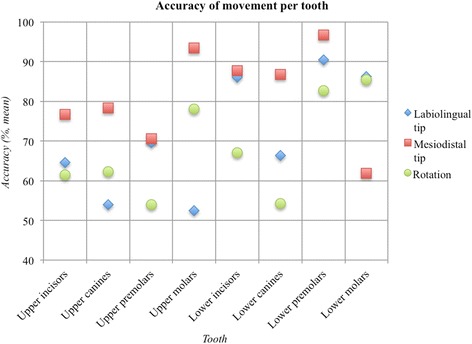
Accuracy of planned movements by tooth type

We found that the most accurate movement achieved by F22 was mesiodistal tipping, whose mean accuracy was 82.5% (SD = 77.4) overall, and 96.7% at the lower premolars (SD = 96.9), closely followed by the upper molars (93.4%, SD = 72.6) and lower incisors (87.7%, SD = 85.9%). Less precise movements were found to be vestibulolingual tipping of the upper molars (52.5%, SD = 53.3) and upper canines (54.0%, SD = 57.2%) and rotation of the upper premolars (54.0%, SD = 54.3) and lower canines (54.2%, SD = 73.9) (Table 6, Fig. 6).

**Table 6:** Mean (%) accuracy of tooth movements achieved using F22

	Vestibulolingual tip			Mesiodistal tip			Rotation		
Tooth	Mean (%)	*n*	SD	Mean (%)	*n*	SD	Mean (%)	*n*	SD
Upper incisors	64.5	28	34.2	76.7	36	57.6	61.5	45	28.5
Upper canines	54.0	16	57.2	78.3	16	50.2	62.3	25	65.6
Upper premolars	69.6	32	80.8	70.6	27	78.2	54.0	29	54.3
Upper molars	52.5	16	53.3	93.4	22	72.6	78	18	61.3
Lower incisors	86.1	35	64.6	87.7	31	85.9	67	51	57.4
Lower canines	66.4	15	55.1	86.7	18	82.3	54.2	25	73.9
Lower premolars	90.4	29	81.5	96.7	33	96.9	82.7	36	138
Lower molars	86.2	7	50.8	61.8	17	82.4	85.4	14	67.4
Total	71.2	178	59.7	81.5	200	75.8	68.1	243	68.3

### Rotation

Rotation movements, especially of rounded teeth like the canines and premolars, are notoriously difficult to achieve with aligners. Indeed, one prospective study [19] conducted on 53 canines in 31 subjects found a mean canine rotation accuracy of 36%. Greater canine rotation accuracy can be achieved with interproximal reduction (IPR), but this only provides an accuracy of 43%, albeit with a lower standard deviation (SD = 22.6%). Another study [14] found a rotation accuracy of 32% at the upper canines and even less at the lower canines (29%), as compared to the upper central (55%) and lower lateral incisors (52%). Moreover, there is an even further significant reduction in the accuracy of upper canine rotation at rotations of greater than 15° (19%; SD = 14.1%; *P* < .05).

Our data confirm that among the lower teeth canine movement is the least accurate. That being said, our predictability percentage was higher than that reported in the literature for other aligner systems (54.2%, SD = 73.9). Furthermore, the F22 aligners achieved an accuracy index not significantly different from 1, i.e. 100% of the prescribed movement, for rotation of the upper molars (0.78, SD = 0.61), lower premolars (0.83, SD = 1.27) and lower molars (0.85, SD = 0.67).

That being said, comparison of all movements achieved by F22 in all tooth categories shows that, with respect to the prescription, the mean rotation of the upper incisors appeared significantly more accurate than the mean rotation of the lower premolars. This is in line with several literature reports on other aligner systems, for example Djeu et al.’s Invisalign study [22], in which they noted that one of the strengths of the system was the ability to correct the rotation of anterior teeth and level the incisor margins. Kravitz [14] also showed that the greatest rotation accuracy is achieved at the upper incisors (mean accuracy 48.8% for central and lateral incisors); Nguyen and Cheng [21] too confirm this finding, reporting a mean incisor rotation of 60%. This parallels our figure of 61.5% (SD = 28.5%), but with F22 aligners, we found that the best rotation accuracy was achieved at the lower molars (85.4%, SD = 67.4) and lower premolars (82.7%, SD = 138)—teeth that were not considered in Kravitz’s analysis—albeit with a high standard deviation.

### Mesiodistal and vestibulolingual tipping

Kravitz’s 2009 study [14] repeated a mean accuracy of 41% for mesiodistal tipping, which was most accurate at both the upper (43%) and lower (49%) lateral incisors; mesiodistal tipping of the upper (35%) and lower (27%) canines and the upper central incisors (39%) was the least accurate. Our F22 results are in line with these findings, in that the least predictable movements achieved in the anterior sector were the upper canines and incisors, although once again, our accuracy scores were markedly higher. Indeed, the mesiodistal tip achieved at neither the upper canines (0.78, SD = 0.5), nor the upper premolars (0.7, SD = 0.78), upper molars (0.93, SD = 1.02), lower incisors (0.88, SD = 0.86), lower canines (0.87, SD = 0.82), lower premolars (0.97, SD = 0.97) or lower molars (0.62, SD = 0.82) was significantly different from 1, considered full achievement of the outcomes predicted by the setup. As regards vestibulolingual tipping, on the other hand, neither the lower incisors (0.86, SD = 0.64), nor the lower premolars (0.9, SD = 0.81) or lower molars (0.86, SD = 0.5) exhibited an accuracy index not significantly different from 1.

The orthodontic movement is a multifactorial issue. There are many parameters that can affect the ability to reach the goal planned in the setup. The crown anatomy, the root length and bone density were taken in consideration in this study dividing the sample into different groups by dental typology. Other parameters like sex and age of the patient could also influence the response to the aligners’ application, as suggested by literature [23]. In addition, the characteristics of the material, thickness, alignment protocol application and staging may affect the efficiency of the orthodontic movement. All these parameters will need to be thoroughly investigated in future research.

There were several limitations to this study. First and foremost, it would have benefitted from a larger sample. Only 16 patients remained after the selection process, giving a potential 448 teeth to be analysed. However, once movements of prescription lower than 2° were excluded, this number fell to 346. Second point, as this is a retrospective study, the cases with complete records are more likely to be those that completed treatment, rather than truly representative of those who started treatment with aligners. This could overestimate the effectiveness of the treatment.

Furthermore, we analysed only three types of tooth movement: rotation, mesiodistal tipping and vestibulolingual tipping; as digital models rather than radiographs were used for measurements, there was no information regarding root position from which to derive torque values. Nevertheless, the method of measurement we used, with the aid of VAM software, did enable us to analyse both anterior and posterior teeth, relying as it did on an “average” occlusal plane, passing through the centroids of the FACC points of all teeth (except for the canines) as a reference. Indeed, this plane is only minimally affected by the tooth movements achieved during treatment. That being said, the occlusal plane cannot be considered entirely stable and, moreover, it is difficult to compare the results of this type of analysis with those in the literature, which derive from superimpositions of the palatine folds and posterior teeth.

Finally, it is worth noting that the study design did not enable us to explore the full potential of F22 aligner treatment. Indeed, complex movements are usually aided by the use of auxiliaries such as elastics or chains, whereas we evaluated outcomes achieved by the F22 Grip Points (attachments) and stripping alone. It is conceivable that in the hands of an experienced orthodontist, with a full array of auxiliaries at their disposal, the accuracy percentages we revealed could be further improved upon.

## Conclusions

Our analysis of the predictability of orthodontic movements that can be achieved using F22 aligners, without auxiliaries, enables us to state that

- The mean accuracy of rotation, mesiodistal tipping and vestibulolingual tipping was 70.6% in the anterior sector and 73.6% across both full arches.
- Mesiodistal tipping was the most predictable movement, reaching a mean accuracy of 82.5%; vestibulolingual tipping and rotation reached 72.9 and 66.8% of the prescribed movement, respectively.
- The least predictable movement was rotation of the lower canines (54.2%), while the most predictable movements were mesiodistal tipping of the upper molars and lower premolars (respectively 93.4 and 96.7%).
- The mean rotation error was significantly greater at the lower canines than at the lower incisors and molars.
- In the upper arch, mesiodistal tipping of the canines, premolars and molars displayed a very high accuracy index, not significantly different from 1. This was also true of vestibulolingual tipping of the molars.
- In the lower arch, the accuracy index was not significantly different from 1 for mesiodistal tipping of all teeth, vestibulolingual tipping of the incisors, premolars and molars, and rotation of the premolars and molars.
- There were no significant differences in the accuracy index between tooth movements, with the exception of upper incisor rotation, which was significantly lower to that achieved at the lower premolars.
- Further research on the topic using such a precise and reproducible means of model superimposition and measurement is required and should involve larger samples in order to shed light on the potential benefits and drawbacks of aligner systems.
